# Scopus Search Analyzer

**DOI:** 10.29173/jchla29752

**Published:** 2024-08-01

**Authors:** Mackenzie Hilton

**Affiliations:** Librarian, CAMH, Toronto, ON, Canada

**Product:** Scopus Search Analyzer

**URL:** https://www.scopus.com/

## Purpose

The purpose of this review is to highlight Scopus’ updated search analyzer tool and to elucidate how this tool can be used for biomedical research.

## Product description

Scopus’ search analyzer provides a quick and efficient way to view journal, author, and affiliation metrics. Analyzing thousands of results in an instant, the search analyzer creates graphical representations that help users visualize the bibliometrics of their search. Pie charts, line graphs, and bar graphs – all colour coded – simplify complex data and allow researchers to identify trends, relevant journals and scholars, and overlap with other subjects.

## Intended users

Scopus’ search analyzer is intended for research at any stage, from background research to systematic reviews.

## Special features

**Fig. 1:**
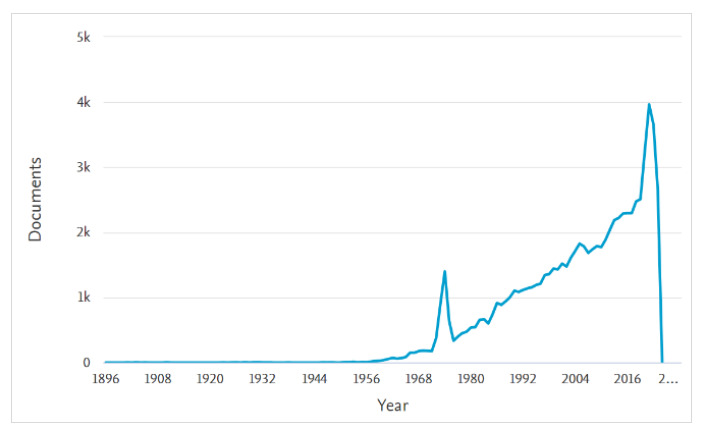
Documents by year

The Documents by year category (Figure 1) displays a graphical representation of the number of documents published on the topic per year. The line graph shows trends and outliers in the research topic. Users can focus on specific years by moving their cursor over the graph. Doing so displays the specific year, and the number of documents from that year in Scopus. Users can also select points on the graph to view the articles from specific years.

**Fig. 2:**
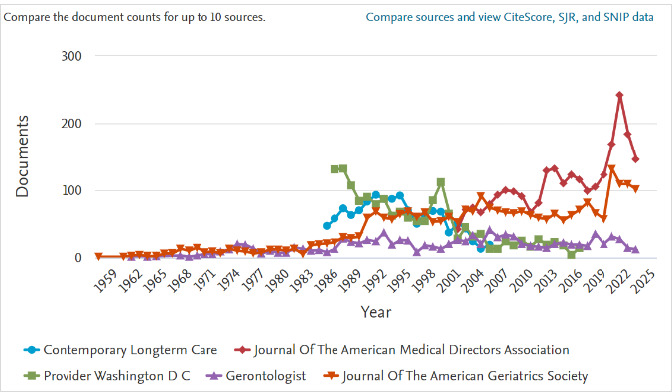
Documents per year by source

Documents per year by source (Figure 2) connects publications with their journals. This can be helpful to identify which journals have published the most on a topic.

**Fig. 3:**
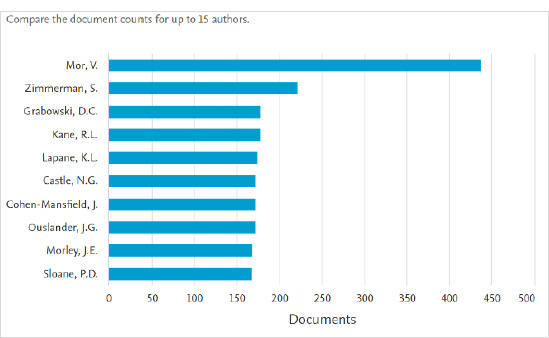
Documents by author

Documents by author (Figure 3) compares the document count for up to 15 authors. The search analyzer defaults to the 15 authors who have published the most on a topic. The number of documents published by an author is shown both numerically beside the author’s name and visually as a bar graph. Users can select other authors to analyze by scrolling through the list on the left and selecting the box beside the author’s name.

**Fig. 4:**
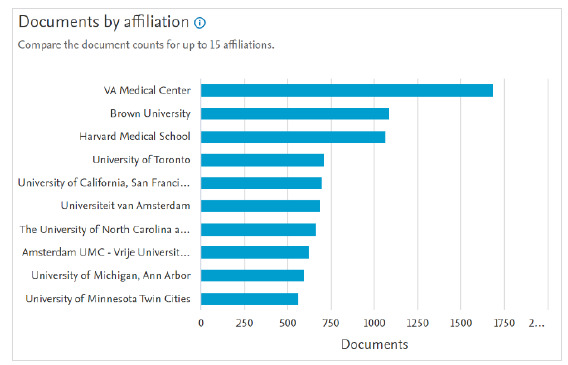
Documents by affiliation

Documents by affiliation (Figure 4) compares the document count for up to 15 affiliations. Users can select which affiliations to analyze from the list to the left of the bar graph. Affiliations are listed alongside their document count. Users can select the number of documents to view that list. Selecting the name of the affiliation will display to users the affiliation metrics, including the number of documents published by that affiliation, the number of authors associated with it, collaborating affiliations, and affiliation hierarchies. Neatly labeled lists and pie charts condense and make accessible otherwise dense information.

**Fig. 5:**
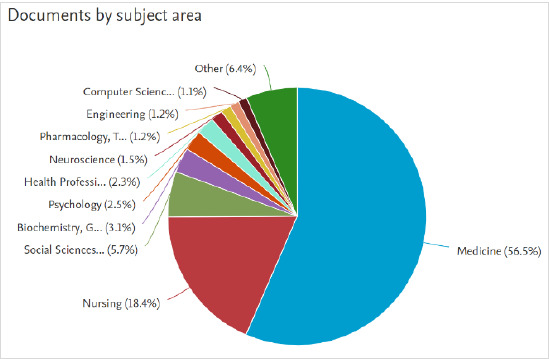
Documents by subject area

Documents by subject area (Figure 5) shows the interdisciplinarity of the topic in the form of a pie chart. Sections of the pie chart are labelled alongside their percentage. Users can hover over specific sections to see the document count for that subject area. This information is also displayed in list form to the left of the pie chart.

## Platform and compatibility

The search analyzer works in any internet browser that supports Scopus. These browsers include Chrome, Firefox, Safari, and Internet Explorer; however, for optimal use, Chrome or Firefox are recommended [1].

## Usability and cost

Scopus requires a subscription to access. Subscriptions can only be purchased by institutions.

The cost of a subscription depends on the number of users within the institution.

## Strengths and weaknesses

### Strengths

- Easy to access, use, and switch between different data sets. • Visualizations are colour-coded and clearly labeled to make identifying information quick and easy.
- Eight categories, with sub-categories, neatly organize author, journal, and affiliation metrics.
- The combination of list views and graphical representations gives users the option to utilize the interface they prefer.
- Users can specify a date range within any category of the search analyzer.
- Bibliometrics can be easily exported, printed, and emailed.

### Weaknesses

- The categories provided are not comprehensive. Users are unable, for example, to analyze search results based on language or open access. Although searches in Scopus can be filtered by these criteria, they cannot be analyzed to produce visualizations and lists.

## Currency

Scopus is updated daily with approximately 11,000 new articles per day [2]. This ensures that the search analyzer has access to the most up to date information. Scopus’ search analyzer has been available since 2015; however, it has undergone significant updates in recent years, having just completed its beta in 2022. Among these updates include better visualization options, such as a variety of colour coded graphs for bibliometrics, an improved user interface, and the ability to specify a date range from any screen within the analyzer.

## Comparison with similar products

The Web of Science provides a search analyzer with comparable features to the one offered by Scopus. These similarities and differences are captured in Table 1.

**Table 1:** Comparison of Scopus’ and Web of Science’s search analyzers

Feature	Scopus	Web of Science
**Account requirements and cost**	Institutional subscription required	Institutional subscription required
**Journal metrics**	Compare up to 10 sources to view: CiteScore SJR SNIP data Users can search for sources by title, publisher, ISSN and/or subject area.	Compared up to 25 sources to view the number of publications
**Author metrics**	Compare the document count for up to 15 authors to view: Number of citations Number of documents H-index (including h-graphs) Documents by source,type,year, and subject Field-weighted citation impacts Co-authors Author position (ie. first author, last author, etc.)	Top 10 authors are shown in a TreeMap chart or bar graph Author impact beamplots are available H-index (no h-graphs) Number of citations Author position
**Affiliation metrics**	Compare the document count for up to 15 affiliations to view: Number of authors affiliated Number of patents Documents of the institution by subject area View affiliation hierarchies View collaborating affiliations	Compare the document count for up to 25 affiliations to view: Institutional affiliations
**Documents by subject area**	Yes	Yes
# of results analyzed*	78 133	22 616
**Specify date option**	Yes	No
**Export options**	Export (CVS and Zip) Exporting as Zip downloads the visualization as .png, .jpg, .pdf, and .svg files Exporting as CVS downloads datasets that can be viewed in Excel Print Email	Download a .jpg of the visualization without any datasets
**User interface**	Visual thumbnails are shown for each category	Drop-down menus
**Visualization options**	Pie charts Line graphs Bar graphs Numerical lists	Bar graphs TreeMap charts**
**Ease of use**	Very easy and intuitive to use	Not as straightforward to use due to the lack of thumbnails, colour- coded graphs. Too many categories (25 in total) makes it difficult to find certain information (affiliation vs affiliations with the department; publication title vs publisher)

* A test search for “nursing homes” was conducted.

** TreeMap charts are not strictly proportional to the value of each entry.

## Conclusion

Scopus’ search analyzer offers researchers a quick and efficient way to view the bibliometrics of a search. The search analyzer provides a visual analysis of a search divided into eight topics. These categories include *document by year, documents by type, documents per year by source, documents by author, documents by affiliation, documents by country/territory, documents by subject area*, and *documents by funding sponsor*. The bar, line, and pie charts provide accurate visualizations of complex data. Unlike similar search analyzers, Scopus, with its simplified user interface, colour-coded graphs, and visual thumbnails allows users to specify date ranges, quickly switch between metrics and data sets, and export datasets.
